# Using the 5-Item Medication Adherence Report Scale (MARS-5) to Screen for Non-adherence to Vitamin and Mineral Supplementation After Bariatric Surgery

**DOI:** 10.1007/s11695-023-07027-x

**Published:** 2024-01-04

**Authors:** Kristina Spetz, Torsten Olbers, Malin Östbring, Zoe Moon, Rob Horne, Ellen Andersson

**Affiliations:** 1 Department of Biomedical and Clinical Sciences, and Department of Surgery, Linköping University, Norrköping, Sweden; 2 Department of Medicine and Optometry, Linnaeus University, Kalmar, and Pharmaceutical Department, Kalmar County Council, Kalmar, Sweden; 3 Centre for Behavioral Medicine, School of Pharmacy, University College London, London, UK

**Keywords:** Adherence, Compliance, Gastric bypass, Sleeve gastrectomy, Bariatric surgery, Vitamin, Mineral, Deficiencies, Obesity

## Abstract

**Introduction:**

Poor adherence to recommended vitamin and mineral supplementation after bariatric surgery is common and challenging for healthcare professionals to identify. There are several questionnaires for self-reporting of adherence to chronic medication, but none has so far been evaluated for assessment of adherence to vitamin and mineral supplementation after bariatric surgery. The aim of this study was to assess the accuracy of the 5-item Medication Adherence Report Scale (MARS-5) in measuring adherence to vitamin and mineral supplementation post bariatric surgery (gastric bypass or sleeve gastrectomy).

**Method:**

The psychometric properties of MARS-5 for vitamin and mineral supplementation were validated in two cohorts: one at 1 year post bariatric surgery (*n* = 120) and the other at 2 years post-surgery (*n* = 211). MARS-5 was compared to pharmacy refill data for vitamin B_12_ and combined calcium/vitamin D as reference.

**Results:**

Correlation analyses demonstrated that the MARS-5 had acceptable validity compared to objectively measured adherence rates from pharmacy refill data (calculated as continuous, multiple-interval measures of medication availability/gaps-coefficient ranged from 0.49 to 0.54). Internal reliability (Cronbach’s *α*) was high: 0.81 and 0.95, respectively. There was a clear ceiling effect where one out of three had a maximum score on MARS-5.

**Conclusion:**

MARS-5 demonstrated acceptable psychometric properties for assessment of adherence to vitamin and mineral supplementation post bariatric surgery.

**Graphical Abstract:**

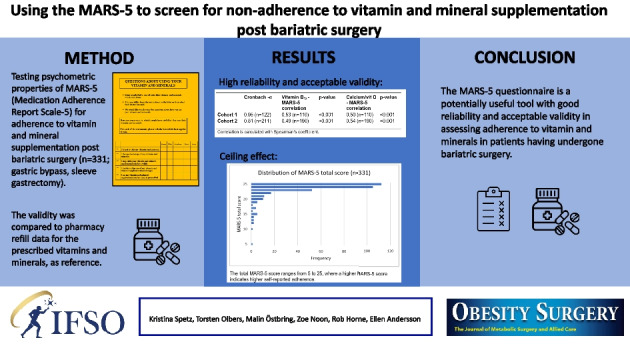

## Background

Bariatric surgery provides long-term weight loss and substantial health gains with a low rate of short-term complications [1, 2]. However, bariatric procedures are associated with long-term risks related to micronutrient deficiencies, such as neuropathy, anemia, and osteoporosis [3–5]. Micronutrient deficiencies are caused by impaired absorption of vitamins and minerals due to the anatomical reconstruction and physiological changes in the gastro-intestinal tract [6]. Lifelong vitamin and mineral supplementation is therefore recommended to prevent development of deficiencies after bariatric surgery [7, 8].

Previous research has shown that 10% of patients discontinue the intended lifelong vitamin and mineral supplementation within the first years after surgery [9–11]. Reasons for poor adherence to vitamin and mineral supplementation are complex but include forgetfulness, experience of side-effects, discomfort with taking or problems with swallowing tablets, financial constraints, and doubts about the need of supplements [10–13].

It is a challenge for healthcare providers to accurately identify patients with poor adherence [14]. To simply ask the patients if they take their supplements is not a reliable method, as patients often overreport adherence due to the social desirability of high adherence [15]. Questions about adherence to medications should be addressed in a non-judgmental way to facilitate accurate reporting [15].

There are several ways to objectively measure adherence to medication such as electronic monitoring, measuring drug concentration in blood or urine, using smart pills, or assessing prescription refill rates [16]. However, in clinical practice, these methods are expensive and time consuming [15]. An inexpensive and easy-to-use alternative way to measure adherence in the clinic is patient questionnaires. Questionnaires on adherence to medication have been designed to reduce the bias of underreporting of poor adherence [14, 16], but to the best of our knowledge, none of these has been evaluated for assessing adherence to vitamin and mineral supplementation after bariatric surgery.

The 5-item Medication Adherence Report Scale (MARS-5) is a widely used instrument for self-reported adherence to medication that has been tested for various pharmaceutical treatments [17]. MARS-5 assesses non-adherence behaviour in a non-threatening and non-judgmental way and has been translated into several languages [18–22]. Existing validations on the accuracy of MARS-5 for different medications cannot be generalized as valid for vitamin and mineral supplementation, as adherence behaviours to vitamin and mineral supplementation may differ from adherence behaviours to pharmaceutical treatment [23].

The aim of this study was to assess the psychometric accuracy of the MARS-5 in measuring adherence to vitamin and mineral supplementation after bariatric surgery.

## Methods

### Study Population

Data in this study was collected from two separate surveys, which have been described in detail elsewhere [9, 24, 25]. The study cohorts include adults undergoing bariatric surgery in the treatment of severe obesity at Vrinnevi Hospital, Norrköping, Sweden. The study participants were consecutively enrolled at routine clinic visits, between May 2017 and January 2019. The bariatric surgical techniques used were laparoscopic Roux-en-Y gastric bypass and sleeve gastrectomy. Exclusion criteria were age ≤18 years and/or inability to read or understand Swedish.

### The MARS-5 Item Questionnaire

The MARS-5 questionnaire consists of five questions on forgetting, changing dosage, stopping, skipping, and taking less medication (Table 3). The score ranges from 5 to 25, where a higher MARS-5 score indicates higher self-reported adherence. One item assesses unintentional non-adherence and four items assess intentional non-adherence. The Swedish translation of MARS-5 has previously been validated for treatment with mood stabilizing medications in bipolar disorder [26].

The study participants completed the MARS-5 questionnaires at the 1-year follow-up visit post-surgery (cohort 1) or got the MARS-5 sent home by letter, with a stamped reply envelope, 2 years post-surgery (cohort 2). A total sample size of 300 participants was deemed sufficient according to recommendations by Comarey and Lee [27], which is also in line with the recommendations of having at least 20 subjects per scale item [28]. Incompletely filled MARS-5 questionnaires were handled as missing data.

### Vitamin and Mineral Supplementation

All patients, regardless of surgical technique, were prescribed a daily lifelong supplementation with combined calcium-vitamin D 500mg/800 IE, and 1 mg vitamin B_12_. At the time of the study, iron was prescribed only to menstruating women and patients with preoperative anemia. Additionally, patients were recommended a daily over-the-counter (OTC) multivitamin tablet.

### Reliability

To evaluate reliability, the internal consistency of MARS-5 was determined. Internal consistency estimates the extent to which items within a scale assess a single construct and is assessed by computing a Cronbach’s *α* coefficient. Values above 0.70 are considered to indicate good internal consistency [23].

### Validity

Criterion-related validity refers to the degree of agreement between scores on a questionnaire and some independent criterion. The validity was evaluated by comparing the scores of MARS-5 with pharmacy refill data, which is a well-established objective adherence measurement. We examined the relationship between MARS-5 and pharmacy refill rates using the Spearman correlation coefficient.

We used pharmacy refill data from the Swedish Prescribed Drug Register, a national register containing all data on prescription refills (dosage, quantity, and date of drug refill) dispensed at pharmacies in Sweden [29]. Adherence rates were calculated for calcium/vitamin D and vitamin B_12_ as continuous, multiple-interval measures of medication availability/gaps (CMA). CMA is defined as the proportion of days covered by a medication during the observation period (40, 41). As the routine prophylactic supplementation after bariatric surgery is unlikely to require total adherence to achieve treatment goals [7], poor adherence was therefore defined as CMA< 50% in this study. Adherence calculations were performed using the AdhereR package, RStudio version 1.1.463 (RStudio Inc., Boston, MA), CMA version 7 [30].

The study participants were also asked if they used non-prescribed complete supplementation designed for patients after bariatric surgery, containing all micronutrients recommended in the Nordic guidelines [3]. Patients reporting use of non-prescribed supplements were excluded from the correlation analysis due to the lack of pharmacy refill data.

### Sensitivity and Specificity

To assess sensitivity and specificity, the MARS-5 sum score was dichotomized into either “adherent” or “non-adherent” using a threshold of < 24 and compared with dichotomized pharmacy refill data as reference. CMA ≤ 50% to either or both vitamin B_12_ and combined calcium/vitamin D was considered non-adherent. Detection of non-adherence was defined as the positive outcome in the calculations of sensitivity and specificity.

### Floor and Ceiling Effects

Floor and ceiling effects were assessed for MARS-5. A floor or ceiling effect means that >15% of patients receive the instrument’s highest or lowest value [22].

### Ethical Approval

The study was approved by the Swedish Ethical Review Authority (2016/1259-31/4; 2017/1406-32; 2017/2101-32; 2017/117-31). All procedures were in accordance with the ethical standards of the Swedish Ethical Review Authority and the 1964 Helsinki declaration and its later amendments or comparable ethical standards. Informed consent was obtained from all individual participants included in the study.

## Results

### Patient Characteristics

Patient characteristics of the two study cohorts are presented in Table 1. Of 402 patients enrolled in the two cohorts, 321 patients (80%) completed MARS-5 and were included in the current study. There was a significant difference in completion rates of MARS-5 between patients with high and low adherence to vitamin and mineral supplementation, measured with pharmacy refill data. In the group with an adherence-rate ≤ 50% to both vitamin B_12_ and combined calcium/vitamin D, 36% did not complete MARS-5 compared to 11% in the group with ≥ 80% adherence to both supplements (*p* < 0.001). There were no statistically significant differences in baseline characteristics between study participants that completed MARS-5 and those who did not.

**Table 1:** Patient characteristics at bariatric surgery

	Cohort 1 *n* = 211	Cohort 2 *n* = 120
Age at surgery (years)	43 ± 11	42 ± 9
Sex: male, female	21.3%, 78.7%	18.3%, 81.7%
Preoperative weight (kg)	115 ± 19	114 ± 18
Preoperative BMI (kg/m^2)^	40 ± 5	40 ± 6
Surgery: gastric bypass, sleeve gastrectomy	88.6%, 11.4%	80.8%, 19.2%
Cardiovascular disease	8.1%	1.7%
Diabetes type 2	14.7%	9.2%
Hypertension	27.0%	20.6%
Education >9 years	88.6%	91.7%

Data are given as mean ± SD or percentage. *BMI*, body mass index. Missing data, data on education in cohort 1 is missing for 3 study participants

### Reliability

The MARS-5 demonstrated high internal consistency, with Cronbach’s *α* coefficients of 0.95 for cohort 1 (*n* = 120) and 0.81 for cohort 2 (*n* = 211) (Table 2).

**Table 2:** Internal consistency for MARS-5, and correlation between MARS-5 and pharmacy refill data for vitamin B_12_ and combined calcium/vitamin D

	Cronbach-*α*	Vitamin B_12_ — MARS-5 correlation	*p*-value	Calcium/vit D — MARS-5 correlation	*p*-value
Cohort 1	0.95 (*n* = 122)	0.53 (*n* = 110)	<0.001	0.50 (*n* = 110)	<0.001
Cohort 2	0.81 (*n* = 211)	0.49 (*n* = 190)	<0.001	0.54 (*n* = 190)	<0.001

Correlation is calculated with Spearman’s coefficient. Cohort 1 completed MARS-5 at 1 year post-surgery and cohort 2 completed MARS-5 2 years post-surgery

### Validity

There was a moderate, significant correlation between the MARS-5 scores and adherence measured with pharmacy refill data (CMA) for vitamin B_12_ (cohort 1: *r* = 0.53, *p* < 0.001 and cohort 2: *r* = 0.49, *p* < 0.001) and combined calcium/vitamin D (cohort 1: *r* = 0.50, *p* < 0.001 and cohort 2: *r* = 0.54, *p* < 0.001) (Table 2). Ten percent (31/321) of the study participants were excluded from correlation analysis due to use of non-prescribed complete supplementation, which is not registered in the Swedish pharmacy refill database.

### Adherence Rates

When adherence was measured with pharmacy refill data, 12% of participants had poor adherence (<50% CMA) to both supplements, while 20% had poor adherence (<50% CMA) to either vitamin B_12_ or combined calcium/vitamin D but acceptable or high adherence to the other supplement. When adherence was measured with MARS-5 with a cutoff score for poor adherence < 24 in MARS-5, 19% were identified as having poor adherence.

### Sensitivity and Specificity

When using a threshold of < 24, the MARS-5 had a sensitivity of 63% and a specificity of 82% in detecting patients with poor adherence to at least one supplement. In MARS-5, the patient is asked about “my vitamins and minerals”, i.e. not each prescribed vitamin and mineral individually. There was a large variability in the MARS-5 score in the group with poor adherence to only one of the prescribed supplements, and therefore an additional analysis was conducted where individuals with poor adherence to only one of the supplements were excluded. In the additional analysis, the MARS-5 had a sensitivity of 72% and a specificity of 82%.

### Distribution of Results

**Fig. 1:**
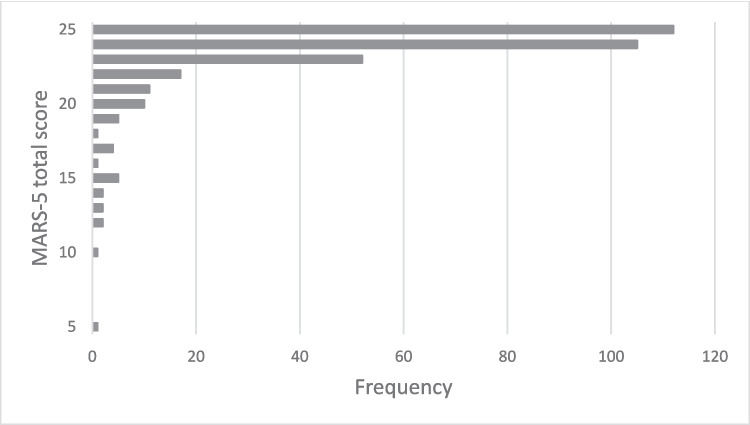
Distribution of MARS-5 (Medication Adherence Report Scale) total score and table notes: The total score ranges from 5 to 25, where a higher MARS-5 score indicates higher self-reported adherence. A total of 331 patients completed MARS-5

The MARS-5 total score is presented in Fig. 1, where a clear ceiling effect can be seen. Mean scores of MARS-5 and its individual items are presented in Table 3.

**Table 3:** Mean scores of Medication Adherence Report Scale (MARS-5) and its individual items

	Cohort 1 mean (SD) *n* = 120	Cohort 2 mean (SD) *n* = 210
Item 1: “I forget to take my vitamin and mineral supplementation”	4.1 (0.8)	4.0 (1.0)
Item 2: “I change the dosage of my vitamin and mineral supplementation”	4.9 (0.4)	4.9 (0.5)
Item 3: “I stop taking my vitamin and mineral supplementation for a while”	4.9 (0.6)	4.7 (0.9)
Item 4: “I decide to skip one of my vitamin and mineral supplementation dosages”	4.9 (0.4)	4.7 (0.9)
Item 5: “I use my vitamin and mineral supplementation less than is prescribed”	4.8 (0.6)	4.7 (0.7)
Total score MARS-5	23.6 (2.0)	23.0 (3.0)

The MARS-5 questionnaire consists of five questions on forgetting, changing dosage, stopping, skipping, and taking less medication. The total score ranges from 5 to 25, where a higher MARS-5 score indicates higher self-reported adherence

## Discussion

To identify poor adherence to vitamin and mineral supplementation after bariatric surgery is crucial for the prevention of complications related to biochemical deficiencies, as about one in five have poor adherence to the supplements. However, detecting patients with poor adherence is challenging in clinical practice. To our knowledge, this study is the first to evaluate a patient questionnaire to assess adherence to vitamin and mineral supplementation after bariatric surgery. The study demonstrates acceptable psychometric properties of MARS-5, suggesting MARS-5 as an easily accessible, inexpensive method for assessing adherence to vitamin and mineral supplementation in clinical follow-up after bariatric surgery.

MARS-5 performed well on the test of reliability, showing good internal consistency with high Cronbach’s *α* which confirms results from previous validations of the MARS-5 for medical treatment of other conditions [17, 18, 22, 26].

In the evaluation of validity of MARS-5 for vitamin and mineral supplementation, MARS-5 was moderately correlated with pharmacy refill data (CMA). Since there is no gold standard, different methods for measuring adherence can be chosen as reference to determine the validity of an adherence questionnaire [15]. Pharmacy refill data is advantageous because it is an objective measurement of medication availability, although it does not tell whether the patient has actually taken the medication [16]. A methodological challenge that may weaken the correlation is that MARS-5 captures more recent medication-taking behaviour while pharmacy refill is a composite measure that averages adherence over time, which in this study is a year [16]. Results show a moderate correlation between MARS-5 and CMA data which is as expected given these limitations.

The ceiling effect makes it challenging to use the MARS-5 to differentiate between degrees of adherence in groups of patients with high adherence rates. This would need to be considered if MARS-5 is to be used when researching adherence to vitamin and mineral supplementation. In a clinical follow-up setting, the ceiling effect is of less importance as the target is to identify patients with poor adherence.

A not completed MARS-5 questionnaire may indicate risk of poor adherence as do low MARS-5 scores, as people with poor adherence measured with pharmacy refill data were less likely to complete the MARS-5 questionnaire.

From a clinical perspective, MARS-5 shows suboptimal sensitivity in identifying patients with poor adherence to vitamin and mineral supplementation. The absence of a gold standard for adherence measurement makes it difficult to set a cutoff for what is an acceptable sensitivity [31, 32]. The sensitivity is in line with results from other studies validating both MARS-5 and other adherence questionnaires, using pharmacy refill data as reference [14, 32, 33]. We found that MARS-5 exhibited higher sensitivity in identifying patients who have poor adherence to all vitamins and minerals, compared to patients who only have poor adherence to either vitamin B_12_ or combined calcium/vitamin D and acceptable or high adherence to the other supplement. A probable explanation is that the questions in the MARS-5 questionnaire refer to multiple treatments (“my vitamin and mineral supplements”) whereas the pharmacy refill data measures adherence to vitamin B_12_ and adherence to calcium/vitamin D separately.

It would be of value to find tools for screening of adherence with greater possibilities of identifying patients with poor adherence to vitamin and mineral supplementation than MARS-5 provides. To combine different methods in assessing adherence may improve measurement accuracy [16]. Future studies need to focus on assessing a multi method approach, possibly combining MARS-5 and routinely collected blood samples on micronutrient status.

Even though the questionnaire does not exhibit optimal sensitivity in identifying patients with poor adherence, other psychometric properties were adequate, and we suggest MARS-5 to be used in clinical follow-up after bariatric surgery. Using MARS-5 in clinical practice improves care by highlighting the important issue of adherence, and identifying those who may be at need of further support, and providing understanding of whether non-adherence may be intentional or unintentional. As seen in previous research, factors affecting adherence to vitamin and mineral supplements are complex [10–13]. Identifying individual barriers and whether the barriers are intentional or unintentional is necessary to be able to support individual patients to improve adherence [15].

A limitation of this study is the limited sample size, and the single-site study concept influences the generalizability of the psychometric evaluation of the MARS-5 questionnaire. However, the operating center provides bariatric surgery for an entire healthcare region including both rural and urban areas. Furthermore, the study population is comparable to the national Swedish bariatric population operated at the time of the study [31].

## Conclusion

Identifying poor adherence to vitamin and mineral supplementation in patients after bariatric surgery is important in order to prevent long-term complications related to micronutrient deficiencies. This study demonstrates that the MARS-5 is a potentially useful tool with good reliability and acceptable validity in assessing adherence to vitamins and minerals in patients having undergone bariatric surgery.
